# Intraoperative primary partial occlusion of the PreserFlo MicroShunt to prevent initial postoperative hypotony

**DOI:** 10.1007/s10792-023-02664-8

**Published:** 2023-03-11

**Authors:** Jan Niklas Lüke, Niklas Reinking, Thomas S. Dietlein, Alexander Haendel, Philip Enders, Alexandra Lappas

**Affiliations:** Department of Ophthalmology, Medical Faculty, University Hospital of Cologne, Kerpener Strasse 62, 50937 Cologne, Germany

**Keywords:** PreserFlo MicroShunt, Innfocus, Trabeculectomy, Occlusion, Filtering stent

## Abstract

**Purpose:**

The aim of the underlying study was to present a new surgical method in PreserFlo MicroShunt surgery for glaucoma. A removable polyamide suture was placed into the lumen of the MicroShunt during implantation to prevent early postoperative hypotony.

**Methods:**

Thirty-one patients undergoing stand-alone glaucoma surgery with implantation of a PreserFlo MicroShunt and an intraluminal occlusion were retrospectively reviewed and compared to a control group without occlusion. Inclusion criteria were diagnosis of primary open-angle glaucoma or secondary open-angle glaucoma due to pseudoexfoliation or pigment dispersion. Patients with a history of filtrating glaucoma surgery were excluded.

**Results:**

IOP decreased from 26.9 ± 6.6 to 18.0 ± 9.5 mmHg at the first postoperative day after PreserFlo MicroShunt implantation. Postoperative removal of the occluding suture resulted in a mean IOP reduction in 11.1 ± 7.6 mmHg. Mean visual acuity was 0.43 ± 0.24 logMAR during the first postoperative examination. The interval with the occluding intraluminal suture in place varied from days to 2–3 weeks. Patients were followed up to 1 year.

**Conclusion:**

Implantation of a PreserFlo MicroShunt combined with an intraluminal suture prevented postoperative hypotony in all patients. Mean postoperative pressure was reduced despite the occluding suture in place.

## Introduction

The PreserFlo MicroShunt (Santen Pharmaceutical Co., Ltd., Osaka, Japan, hereinafter referred to as MicroShunt) is an ab-externo filtering stent for the treatment of glaucoma by lowering the intraocular pressure (IOP) [1].

Consistent lowering of IOP can still be considered the most important treatment approach to prevent the progression of glaucoma [2]. The treatment of glaucoma is based on two major strategies. Depending on the level of IOP, the severity of glaucoma and intolerance to local antiglaucomatous agents, medical treatment or surgical interventions are established therapies [2].

The MicroShunt is often classified as a microinvasive glaucoma surgical procedure (MIGS)[3]. Previously, developed MIGS procedures (e.g. iStent) were primarily designed for patients with mild to moderate glaucoma, as postoperative IOP reduction has been insufficient for severe glaucoma [4].

The MicroShunt is an ab-externo procedure, which drains aqueous humor from the anterior chamber to a sub-Tenon/subconjunctival space forming a conjunctival bleb. In contrast to established procedures like trabeculectomy with mitomycin C, treatment with MIGS aims to minimize surgical trauma and associated risk for the patient [5]. Additionally, increased standardization is thought to reduce the need for postoperative interventions in case of hypotony or early bleb failure.

The material used to design the MicroShunt is styrene-*block*-isobutylene-*block*-styrene (SIBS), which has been shown to be biocompatible in preclinical studies and did not lead to any inflammatory reaction [6]. The MicroShunt has a centrally located fin to prevent dislocation. Different lumen diameters have been examined in an animal model. The lumen diameter of 70 µm with a length of 8.5 mm has been found to have the lowest risk of chronic hypotony in the rabbit model, with sufficient pressure reduction [7]. However, initial clinical observations showed a relevant share of patients with early postoperative hypotony within the first postoperative days (28.9% *n* = 395 patients [8]). Hypotony can lead to severe complications like reduced visual acuity, intraocular hemorrhage and choroidal hemorrhage. To temporarily reduce the intraluminal flow of the aqueous humor through the MicroShunt, an intraluminal polyamide filament was used for partial occlusion of the MicroShunt.

## Materials and methods

### Study design

All patients undergoing glaucoma surgery with stand-alone implantation of a MicroShunt medical device at the Department of Ophthalmology, University of Cologne, Germany between 01.07.2019 and 17.12.2020 were retrospectively reviewed.

Inclusion criteria to this retrospective analysis were diagnosis of primary open-angle glaucoma or secondary open-angle glaucoma due to pseudoexfoliation or pigment dispersion. After initial observations of several hypotonies after surgery without intraluminal occlusion, these patients were compared to a group with intraluminal occlusion with a Dafilon 8.0 suture (material: polyamide; diameter 0.04 mm, B. Braun Melsungen AG, Germany). Patients were either phakic or pseudophakic. Patients with previous glaucoma surgery were included in the analysis excluding patients with a history of glaucoma drainage device surgery, any other filtrating surgery or vitrectomy. A maximum of one eye was included per patient.

Additional data including the postoperative follow-up were derived from the patient’s medical records. All patients included in this analysis received treatment from one of the department’s most experienced glaucoma surgeons (AL, TD).

### Patients and methods

Preoperative and postoperative follow-up examinations were reviewed and included slit lamp examination, fundus examination, perimetry, ultrasound sonography when indicated, measurement of IOP by iCare rebound tonometry (iCare, Finland Oy, Finland) and BCVA (best corrected visual acuity). Postoperative examinations were carried out in our department at different time points and were reviewed up to one-year postoperatively. Outcome measures were pre- and postoperative IOP, visual acuity, complications and local medication. Patients were subdivided into four groups based on the time of suture removal (group one: < 24 h; group two: day 2–4; group three: day 5–14; group four: > 14 days). These patients were compared to the control group in which no intraluminal filament was used (*n* = 18).

All included patients were followed up for one-year postoperatively. Examinations were performed on the first postoperative day, within the first 2 weeks, after 6–8 weeks and one-year postoperatively.

### Surgical technique

In subconjunctival anesthesia or general anesthesia, a subconjunctival and sub-Tenon flap was created using a limbal opening. The surgery was performed according to the recommendations of the manufacturer: Three mitomycin-C-soaked sponges were placed in the sub-Tenon space for three minutes. A marker was used to mark the primary site of the scleral incision at 3 mm peripherally from the conjunctival limbus. Lamellar scelerectomy was performed using a triangular knife with a 1 mm width according to the manufacturer´s specifications. Following this incision, a 25-gauge needle created a transscleral tunnel between the prepared scleral pocket for the fins of the MicroShunt and the anterior chamber. The MicroShunt was inserted into the tunnel with wedged fins in the scleral pocket. Regular aqueous humor flow through the shunt was confirmed by injecting balanced salt solution into the anterior chamber and visualizing a drop of aqueous humor by a sponge at the distal end of the lumen.

**Fig. 1:**
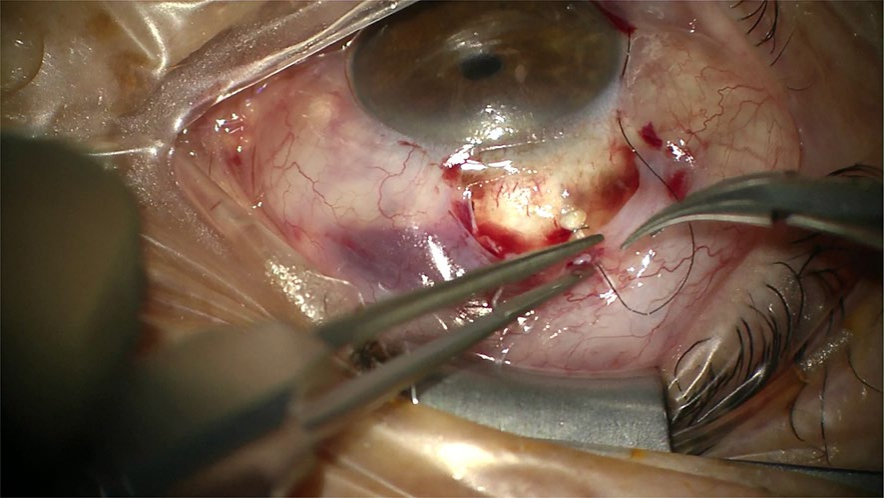
Intraoperative cannulization: After correct placement of the MicroShunt, a Dafilon 8–0 suture is introduced intraluminally. The suture is conducted through an intrascleral space onto the conjunctiva for fixation

In the occlusion group, an 8–0 beveled polyamide monofil suture was then inserted into the lumen by a 25-gauge needle to achieve partial occlusion (Fig. 1). The diameter of the suture was chosen considering the diameter of the lumen of the MicroShunt: According to Hagen-Poiseuille's equation, the volume flow rate is proportional to the radius of the tube to the fourth power. It was assumed that a reduction in diameter of 40 µm would limit the flow to a minimum ([(70–40 µm)/70 µm]^4^ = 3.37%) of initial flow in case of a completely introduced suture. As a complete blockage would reduce the volume flow to less than 5%, the filament was introduced just about one third to one fourth of the length of the shunt.

**Fig. 2:**
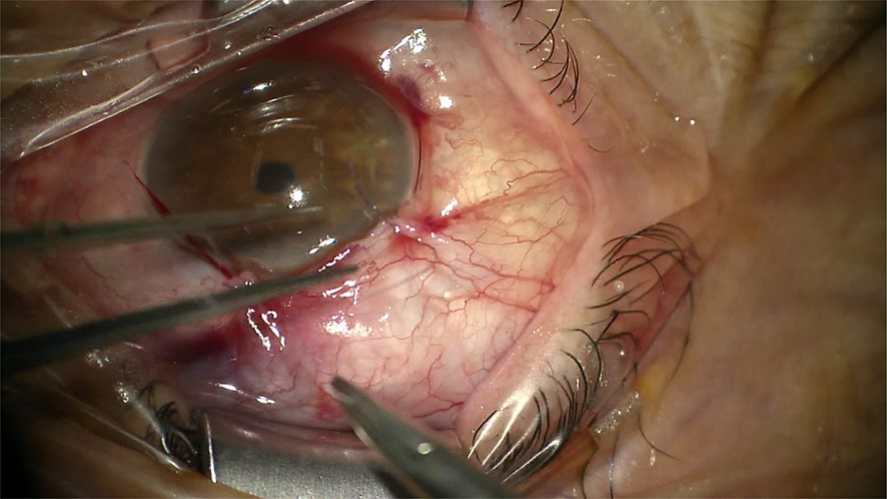
Suture is placed perilimbally parallel to the limbus after closure of the conjunctiva to ensure sufficient reachibility for removal

**Fig. 3:**
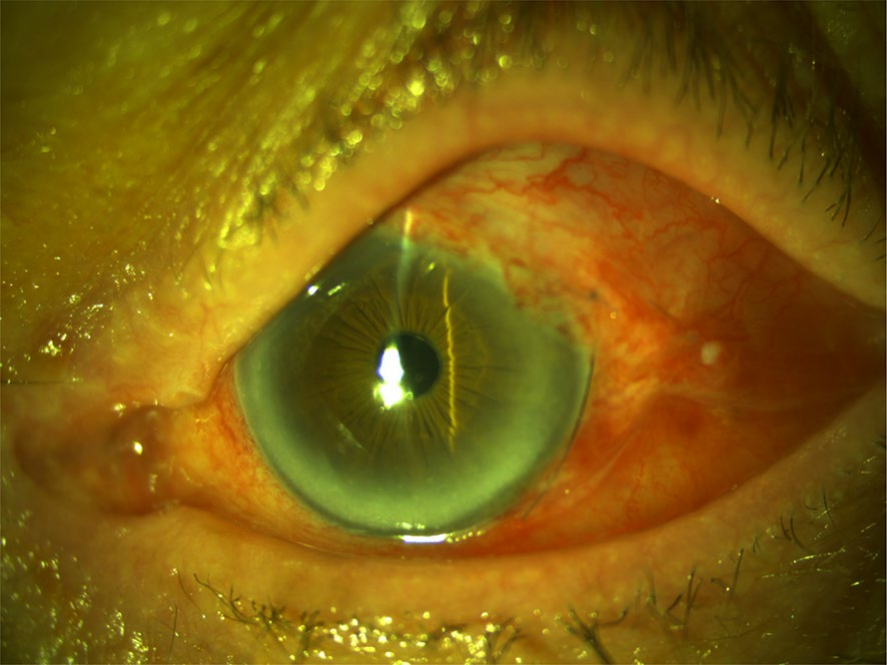
Slit lamp photograph captured on the fifth postoperative day. The intracameral superior located MicroShunt is visible. Corneal endothelium was not touched by the MicroShunt. The suture is located temporally on the limbus and can be removed easily with a forceps after local anesthesia. Before suture removal, a bleb can be observed

When correct placement of the distal end of the MicroShunt could be ensured, the Tenon—and the conjunctival flap were closed separately using absorbable sutures (Vicryl 8.0). The intraluminally placed filament was externalized through the sclera onto the conjunctiva *without placing a knot* at the distal end to allow later removal (Figs. 2 and 3). Suture removal out of the lumen was performed during slit lamp examination with fine forceps after the application of anesthetic eye drops.

### Ethics and statistics

According to regulations of the professional code for Physicians and after consultation with the Ethics Committee of the University of Cologne, an ethical review of the analysis was not required due to the retrospective nature of the study. Values are presented as mean ± standard deviation of the mean (SD). As a nonparametric comparison, Spearman’s rank correlation test was used to compare parameters in case of not normal distributed parameters. Statistical significance was set at *p* < 0.05. All analyses and data presentations were performed with Excel (Microsoft Office Excel 2016, Californian, USA), SPSS v. 22 (IBM Chicago, Illinois, USA) and GraphPad Software (GraphPad Prism 7, Inc, La Jolla, USA).

## Results

Thirty-one patients who underwent MicroShunt implantation with an occluding monofil suture were included in the analysis. Eighteen patients underwent surgery without an intraluminal suture. Details of epidemiological data are illustrated in Table 1.

**Table 1:** Epidemiological and baseline data SD, standard deviation; BCVA, best corrected visual acuity; IOP, intraocular pressure; SLT, selective laser trabeculopasty

	Study eyes (*n* = 31)	Control group (*n* = 18)
*Gender n (%)*		
Men	10 (32.3)	6 (33.3)
Women	21 (67.7)	12 (66.6)
*Age (years)*		
Mean ± SD	75.0 ± 8.5	72.7 ± 9.6
Median	76	73
Range	57–87	60–87
*Glaucoma diagnosis*		
Primary open-angle glaucoma	28 (90.3)	14 (77.7)
Pseudoexfoliation glaucoma	3 (9.7)	4 (22.2)
*Eye n (%)*		
Left	17 (54.8)	12 (66.6)
Right	14 (45.2)	6 (33.3)
*Lens status at baseline n (%)*		
Phakic	15 (48.4)	12 (66.6)
Pseudophakic	16 (51.6)	6 (33.3)
*BCVA at baseline in logMAR*		
Mean ± SD	0.24 ± 0.28	0.33 ± 0.47
Median	0.15	0
Range	0–1.3	0–1
*IOP at baseline (mmHg)*		
Mean ± SD	26.9 ± 6.6	21.5 ± 7.1
Median	26	21.5
Range	14.0–46.0	12–37
*Number of topical and systemical antiglaucomatous medications at baseline*		
Mean ± SD	4.5 ± 2.0	5.1 ± 1.2
Median	5	5
Range	0 to 8	3 to 7
*Other glaucoma surgery n (%)*		
SLT	9 (29.0)	1 (5.5)
Angle surgery (Ab-interno trabectome)	3 (9.7)	1 (5.5)

Follow-up examinations were carried out one-day postoperatively and every day until patients were discharged, 14-days postoperatively, 6–8 weeks postoperatively and 1 year after surgery.

### Morphological results

When split lamp examination was performed on the first two-days postoperatively, a filtering bleb was visible in all cases. The MicroShunt was placed correctly without corneal endothelial touch. At this early postoperative examination, the occluding suture was still located in the intended position in all patients.

### Postoperative complications

In our series, we observed only one case (3.2%) of postoperative hypotony (5 mmHg) when an intraluminal filament was introduced, whereas 4 patients (22.2%) developed postoperative hypotony when no filament was introduced (*n* = 3 with 5 mmHg and *n* = 1 with 3 mmHg).

No severe postoperative hemorrhage, inflammation or other severe complications were observed in either group.

### Intraocular pressure and visual acuity

In patients, who underwent surgery with an occluding intraluminal filament, preoperative mean IOP was 26.9 ± 6.6 mmHg. Preoperative IOP in the control group was 21.5 ± 7.5 mmHg. The preoperative IOP of both groups differed significantly (*p* < 0.01).

The mean IOP on the first postoperative day was 18.0 ± 9.5 mmHg in the occlusion group, showing a mean difference in IOP of 8.0 ± 10.4 mmHg (29.7% reduction). The control group showed a significantly higher relative mean difference in IOP (12.1 ± 8.9 mmHg; 56.1% reduction; *p* < 0.05) with a postoperative mean IOP of 9.4 ± 6.2 mmHg. The absolute mean values of IOP on the first postoperative day were significantly lower in the control group compared with the occlusion group (*p* < 0.001). No significance was detected in the absolute difference between preoperative and postoperative IOP values when comparing the two groups.

Values of BCVA can be found in Table 1.

### Suture removal

The intraluminal occluding filament was removed at different time points postoperatively. The treating surgeon decided when to remove the occluding filament based on the morphology of the filtering bleb, the IOP level and the individual risks of the patient. In case of a postoperative IOP above 20 mmHg, the occluding filament was always removed (Table 2).

**Table 2:** Pressure values pre- and post-suture removal together with the belonging Delta are presented in mmHg compared to the control group

Interval	Occlusion group (*n* = 31)			
	IOP before suture removal	IOP level after suture removal	Difference	Control group (n = 18)
0.–1. Day	22 ± 9.7	9.6 ± 3.9	12.4 ± 6.9	9.4 ± 6.2
2.–4. Day	24.6 ± 9.7	9.6 ± 3.9	14.3 ± 10.2	
5.–14.- Day	19.1 ± 5.1	9.3 ± 4.1	9.9 ± 5.0	9.2 ± 3.0
14 Days	15.0 ± 5.2	10.5 ± 2.5	4.5 ± 3.7	
6–8 weeks	–	11.8 ± 4.8	–	14.3 ± 6.7
12 month	–	13.1 ± 3.4	–	14.2 ± 2.6

Occlusion group 1 included 10 of 31 patients, in which the occluding suture was removed within the first 24 postoperative hours. In these patients, the mean IOP before the removal was 22.0 ± 9.7 mmHg.

Occlusion group 2 included 8 of 31 patients, in which removal took place between the second and the fourth postoperative day. IOP levels before removal were comparable to the first group (24.6 ± 9.7 mmHg).

In occlusion group 3 in 8 of 31 patients, the suture was removed between the fifth and the fourteenth postoperative day with an initial preoperative IOP level of 19.1 ± 5.1 mmHg.

In occlusion group 4 (5 cases), the filament was removed at a later point in time (later than the fourteenth postoperative day). The late removal of the occluding filament was either due to low IOP values (< 10 mmHg, *n* = 2) or because patients postponed their scheduled follow-up examinations (*n* = 3). All details can be found in Table 2.

The mean postoperative IOP difference before and after the occluding suture removal within the four occlusion groups was 4.5 ± 3.7 mmHg (> 14 postoperative days, group 4), 9.9 ± 5.0 mmHg (postoperative day 5–14, group 3), 12.4 ± 6.9 mmHg (postoperative day 0–1, group 1) and 14.3 ± 10.2 mmHg (postoperative day 2–4, group 2), respectively.

When the occluding suture removal was performed within the first two-days postoperatively a functional filtering bleb was always visible (Fig. 3). Even in cases with longer intervals of intraluminal occlusion (> 14 days) and an IOP of < 20 mmHg, a bleb was always visible indicating filtration.

As the final IOP seemed to differ between the four occlusion groups, we analyzed whether there was a correlation between the postoperative time point of the occluding filament removal and the final postoperative IOP. The IOP difference after removal of the occluding suture was dependent on the postoperative time point of the suture removal: Correlating the postoperative time point of suture removal with the respective IOP difference in all groups revealed a significant negative correlation (*p* = 0,053; *r* = − 0.357). The later the occluding suture was removed, the lower the IOP difference was. This implies that deferring the suture removal leads to a lower IOP reduction.

This result proved to be independent of preoperative IOP levels in the occlusion group: We compared preoperative and early postoperative IOP and found that IOP at day one after surgery did not significantly correlate with preoperatively measured IOP in any of the 4 groups (*p* = 0.121).

Follow-up examinations after removal of the occluding filament confirmed a significantly lowered mean final IOP: Mean postoperative IOP of groups 1–4 was 10.3 ± 3.9 mmHg, 28 ± 8 days after surgery and 11.5 ± 4.7 mmHg, 64 ± 13 days after surgery, without any IOP lowering medication.

Subdivided into the four occlusion groups and the control group, the follow-up examination carried out between 4 and 8 weeks postoperatively showed comparable final IOP values in each group. (group 1: 12.2 ± 4.5 mmHg; group 2: 12.0 ± 5.5 mmHg; group 3: 9.9 ± 5.1 mmHg; group 4: 10.2 ± 3.0 mmHg; control group: 14.3 ± 6.7 mmHg).

No significant difference was found between the IOP of the occlusion group 1–4 (13.1 ± 3.4 mmHg) and the control group (14.2 ± 2.6 mmHg) one year after surgery.

## Medication

Preoperatively, the mean score of local medication agents was 4.5 ± 2.0 (Table 3). Postoperatively, no IOP-lowering local medication was documented (4–8 weeks postoperatively). Standard postoperative treatment included 1.3 mg/ml dexamethasone dihydrogen phosphate disodium and 3 mg/ml ofloxacin eye drops (both unpreserved, 4 times daily).

**Table 3:** Medication score: Modificated according to Klewin et al. (2019)

Agent	Score
Timolol 0,5% Acetazolamid 750 mg^3^	1
Brimonidin/Clonidin/Apraclonidin	1
Dorzolamid/Brinzolamid	1
Pilocarpin 1%	1
Pilocarpin 2–4%	2
Carbachol 0,75–3%	2
Bimatoprost/Latanoprost/Tafluprost/Travoprost	2
Acetazolamid 125 mg	1
Acetazolamid 250–500 mg	2
Acetazolamid > 750 mg	4

## Postoperative reinterventions

At the follow-up examination after one year, the IOP values were comparable, with no significant difference between the occlusion group (13.1 ± 3.4 mmHg) and the control group (14.2 ± 2.6 mmHg). In both the occlusion group and the control group, three IOP lowering revision procedures were necessary within the first postoperative year (control group: 16.6%; occlusion group: 9.67%). In each group, one needling procedure and two revision procedures were necessary due to increased IOP.

No revision due to hypotony was required in the occlusion group. Due to initial hypotony, intracameral injection of sodium hyaluronate was performed in one case 2 days after MicroShunt implantation without intraluminal filament.

## Discussion

In this study, we evaluated glaucoma patients after PreserFlo MicroShunt surgery with or without temporary occluding intraluminal filaments. The occluding filament was introduced to avoid postoperative hypotony.

Early postoperative hypotony and uveal effusion are common complications after trabeculectomy and MIGS. Cases of hypotony have also been documented in our clinic and the literature when using the PreserFlo MicroShunt, although the incidence seems to be lower compared with trabeculectomy [10].

We were able to show that the occluding filament could successfully prevent early postoperative hypotony with a low final IOP up to one-year postoperatively.

On the first postoperative day, mean IOP was 18.0 ± 9.5 mmHg in the occlusion group and 9.4 ± 6.2 mmHg in the control group. Using an occluding filament resulted in a higher yet normal IOP in the early postoperative phase.

After removal of the occluding filament, the mean postoperative IOP in the occlusion group was 10.3 ± 3.9 mmHg (28 ± 8 days after surgery) and 11.5 ± 4.7 mmHg (64 ± 13 days after surgery) in the control group, without application of any IOP lowering medication. Follow-up after up to one year revealed comparable results in the occlusion and the control group.

One additional factor that might have influenced the higher early postoperative IOP in the occlusion group might be the preoperative IOP. The preoperative IOP values were significantly higher in the occlusion group compared to the non-occlusion group (26.9 ± 6.6 mmHg, versus 21.5 ± 7.5 mmHg).

This difference could be linked to the retrospective nature of our study as patients with higher preoperative IOP were more likely to be treated with an occluding suture to avoid a high IOP drop after surgery. Thus, the significant early difference in the IOP between the occlusion group and control group could not be validated clearly in our study. However, there is a point to be made in favor of an actual IOP modulation caused by our intraluminal filament, as we could observe that the removal of the occlusion was time-dependent: The earlier the occluding filament was removed the lower the resulting IOP would be. Moreover, the long-term final IOP in the control group and the occlusion group was similar despite the early postoperative differences, and the final postoperative IOP was statistically independent of the preoperative IOP. As the final filtering effect was comparable in both groups and the IOP modification by the occluding filament was limited to the early postoperative period, a causal effect of the filament is highly probable. Our results show that the occluding suture seems to provide a transient resistance to the outflow for as long as 2–3 weeks postoperatively.

In particular, the relative IOP drop due to surgery can therefore only be used for interpretation to a limited extent. However, since surgery was performed in a standardized manner by the same surgeons and the intraluminal filament was only inserted to one third, the higher values in the occlusion group on the first postoperative day can rather be attributed to the intraluminal filament. In addition, the postoperative IOP value was independent of the IOP values before surgery.

In our series, when an intraluminal suture was introduced, we observed only one case of transient hypotony (5 mmHg).

In general, the PreserFlo implant was associated with a lower incidence of hypotony compared with trabeculectomy, as well as XEN [10]. A meta-analysis including 1213 PreserFlo MicroShunt surgeries showed rates between 1.7 and 39% (median 11.1%) for postoperative hypotony and choroidal effusion/detachment rates of 2.0–12.9% (median 8.9%) [11]. Despite the high rate of transient hypotony reported in some studies, the requirement for reintervention considering anterior chamber reconstruction is substantially low (2%), indicating that postoperative hypotony is often self-limiting [10].

Especially in patients with anticoagulant therapy, postoperative hypotony bears a significant risk of hemorrhagic choroidal detachment, which has recently been described in one case 12 days after MicroShunt implantation [12]. Since safety aspects often represent a major factor when determining the indication for MicroShunt implantation, a further risk reduction for hypotony through the placement of an intraluminal filament appears to be quite sensible.

Suture removal at different time points postoperatively did not lead to significantly different IOP one year after MicroShunt implantation. MicroShunt implantation combined with an intraluminal occluding suture resulted in low IOP after up to one year in all patients.

Partial occlusions of the lumen of the MicroShunt seem to be an effective way to reach postoperative normotonic to slightly hypertonic IOP values.

In most cases, the suture was introduced just about one third to one fourth of the length of the shunt, as it was assumed, that a complete blockage would reduce the volume flow to less than 5%. This method allowed the formation of a prominent bleb in all cases with the intraluminal occlusive suture still in place due to flow around the shunt.

The reintervention rate due to increased pressure in the first year after MicroShunt implantation was not increased in the occlusion group in our study (9.67%) compared with data from the meta-analysis (1.2–15.6%) [11].

As preoperatively high IOP values did not lead to elevated final postoperative IOP levels, the implantation of the MicroShunt with intraluminal blockage seemed to provide an independent lowering of IOP, even with an occluding filament. This means that it has proven to be an effective and safe method of treating even severe forms of open-angle glaucoma uncontrollable by local antiglaucomatous drugs.

Interestingly, it could also be observed that the final IOP after the removal of the suture was dependent on the point in time of its removal. A higher reduction in IOP could be achieved by an early removal, a later removal resulted in a lower reduction in IOP. Due to this negative correlation, removal of the occluding filament could be prolonged for 2–3 weeks to achieve only a moderate IOP reduction.

The date of suture removal could be determined not only by the postoperative IOP but also by observing the filtering bleb. After longer intervals of intraluminal blockage (> 14 days), a bleb was still visible, showing that the filtering function could be preserved during this period. Presumably, the flow of intracameral fluid around the shunt, but not intraluminal flow seems to be the reason for preserved filtering function. It seems that long periods without suture removal can be beneficial to prevent hypotony in these cases.

Similar procedures to our intraluminal filament technique with the PreserFlo MicroShunt have been described with the Ahmed glaucoma valve as an ab-interno tube occlusion technique. Removable tube-occluding sutures have been used to treat cases of hypotony maculopathy after Ahmed glaucoma valve implantation. In these cases, a 5–0 polypropylene suture (Prolene; Ethicon) was introduced intraluminally and an external cauterization was performed for fixation [13, 14].

In our series, none of the 31 performed MicroShunt implantations showed any severe complications. Although the occluding filament connected the extraocular to the intracameral space, no moderate or severe inflammation or endophthalmitis occurred.

As a retrospective study, the present study is limited in many respects. Due to multimorbidity and age, many patients did not choose the tertiary care center for their follow-up examinations. This caused the number of patients to be small and the follow-up period to be limited to one year. As a further result, systematic bias occurs: Patients who required reintervention returned for follow-up examinations. Patients who showed regular IOP values in the long-term course were less likely to appear at a tertiary care center for follow-up examinations and could therefore not be included. Nevertheless, the reintervention rates of the occlusion group are within the range of the rates reported in the literature.

## Conclusion

Hypotony after MicroShunt implantation is an adverse effect, which can cause severe complications like hemorrhagic choroidal detachment after surgery. In our series, hypotony could be successfully prevented by transiently occluding the lumen of the PreserFlo MicroShunt with a polyamide monofil suture. Removal of the intraluminal suture up to 2–3 weeks postoperatively resulted in an immediate lower IOP. After 4–8 weeks up to one year, a low final IOP could be reached. No additional IOP-lowering medication was needed in any patient after a one-year follow-up in either the occlusion group or the control group.

## Abbreviations

IOP: Intraocular pressure
MIGS: Micro-invasive glaucoma surgery procedure
SIBS: Styrene-block-isobutylene-block-styrene
BCVA: Best corrected visual acuity
SD: Standard deviation of mean
